# Site-Specific Labeling of the Type 1 Ryanodine Receptor Using Biarsenical Fluorophores Targeted to Engineered Tetracysteine Motifs

**DOI:** 10.1371/journal.pone.0064686

**Published:** 2013-05-28

**Authors:** James D. Fessenden, Mohana Mahalingam

**Affiliations:** Department of Anesthesia, Perioperative and Pain Medicine, Brigham and Women's Hospital, Boston, Massachusetts, United States of America; CNR, Italy

## Abstract

The type 1 ryanodine receptor (RyR1) is an intracellular Ca^2+^ release channel that mediates skeletal muscle excitation contraction coupling. While the overall shape of RyR1 has been elucidated using cryo electron microscopic reconstructions, fine structural details remain elusive. To better understand the structure of RyR1, we have previously used a cell-based fluorescence resonance energy transfer (FRET) method using a fused green fluorescent protein (GFP) donor and a fluorescent acceptor, Cy3NTA that binds specifically to short poly-histidine ‘tags’ engineered into RyR1. However, the need to permeabilize cells to allow Cy3NTA entry as well as the noncovalent binding of Cy3NTA to the His tag limits future applications of this technique for studying conformational changes of the RyR. To overcome these problems, we used a dodecapeptide sequence containing a tetracysteine (Tc) motif to target the biarsenical fluorophores, FlAsH and ReAsH to RyR1. These compounds freely cross intact cell membranes where they then bind covalently to the tetracysteine motif. First, we used this system to conduct FRET measurements in intact cells by fusing a yellow fluorescent protein (YFP) FRET donor to the N-terminus of RyR1 and then targeting the FRET acceptor, ReAsH to an adjacent Tc tag. Moderate energy transfer (∼33%) was observed whereas ReAsH incubation of a YFPRyR1 fusion protein lacking the Tc tag resulted in no detectable FRET. We also developed a FRET-based system that did not require RyR fluorescent protein fusions by labeling N-terminal Tc-tagged RyR1 with FlAsH, a FRET donor and then targeting the FRET acceptor Cy3NTA to an adjacent decahistidine (His_10_) tag. A high degree of energy transfer (∼66%) indicated proper binding of both compounds to these unique recognition sequences in RyR1. Thus, these two systems should provide unprecedented flexibility in future FRET-based structural determinations of RyR1.

## Introduction

A complete understanding of a protein's function in both physiological and pathophysiological states requires an accurate understanding of its structure. A key experimental tool for monitoring changes in protein structure is through measurement of fluorescence energy resonance transfer (FRET) between donor and acceptor fluorophores site-specifically targeted to the protein [1]. Since FRET is highly dependent on the distance between these fluorophores, these measurements can resolve distance changes in the subnanometer range, thus providing a means to quantify subtle changes in protein conformation that reflect its structural dynamics.

FRET measurements have begun to play an important role in understanding the structure of the ryanodine receptor (RyR), the largest ion channel known, which releases Ca^2+^ during excitation-contraction coupling in striated muscles [2]. For example, the position and orientation of key regulatory proteins on the RyR such as FK506 binding protein 12 kDa (FKBP12) and calmodulin (CaM) have been elucidated using FRET measurements [3], [4], [5]. In addition, small changes in FRET efficiencies between fluorescent proteins fused into the RyR have been observed that may reflect agonist-induced conformational changes within the RyR [6], [7], [8].

A central challenge in FRET-based structural measurements of the RyR is specific targeting of donor and acceptor molecules to this massive protein. This challenge has been overcome in several ways including the use of fluorescently tagged RyR-associated proteins [3], as well as fluorescent protein fusions within the RyR primary sequence [6]. Our approach has been to use the site-specific labeling reagent Cy3NTA [9], which binds to short poly-histidine “tags” engineered into the primary sequence of RyR1 [10], [11]. Cy3NTA can then act as a FRET acceptor in conjunction with fused GFP, which acts as a FRET donor. However, this method has several drawbacks including the need to permeabilize cells to permit entry of Cy3NTA and the fact that Cy3NTA binding is noncovalent. Both of these factors reduce the ability to make dynamic conformational measurements of the RyR in living cells using this method.

These problems can potentially be overcome through the use of biarsenical labeling reagents that covalently bind to tetracysteine (Tc) tags [12], [13]. The biarsenicals FlAsH and ReAsH [13] can freely cross cell membranes to bind covalently to the short recognition sequence, CCPGCC, which forms a hairpin binding loop when inserted into a target protein [14]. Binding stability can be improved by addition of flanking sequences around the central CCPGCC motif resulting in the optimized recognition sequence, FLNCCPGCCMEP [15] to which biarsenicals can bind with nanomolar affinity. Upon binding to this motif, FlAsH and ReAsH become fluorescent with spectral properties similar to YFP and dsRed, respectively. Labeling by these compounds can then readily be detected via epifluorescence microscopy in intact living cells [16].

In this report, we have tested the feasibility of using FlAsH and ReAsH for FRET-based structural determinations of the RyR. We found that the RyR can be labeled with either FlAsH or ReAsH when a Tc Tag is fused to the N-terminus of the protein. In addition, these molecules can act either as a FRET donor (in the case of FlAsH) in conjunction with the FRET acceptor, Cy3NTA, or as a FRET acceptor (in the case of ReAsH) in conjunction with a fused YFP FRET donor. We also examined the possibility of orthogonal labeling studies with both FlAsH and ReAsH targeted to low and high affinity Tc tags on the RyR, which has been suggested in recent studies [17].

## Materials and Methods

### Ethics Statement

This study utilized the human embryonic kidney cell line (HEK-293T) acquired from the American Type Culture Collection (ATCC). The Institutional Biosafety Committee at Brigham and Women's Hospital approved the use of these cells for our studies.

### cDNA Cloning

The cDNA encoding the yellow fluorescent protein (YFP) variant, citrine [18] (generously provided by Dr. Roger Tsien, UC San Diego) was fused to the N-terminus of RyR1 to create construct (YFP)RyR1. We then attached either the core CCPGCC sequence or the optimized Tc motif, FLNCCPGCCMEP to the N-terminus of this construct to create CC(YFP)RyR1 and FLN(YFP)RyR1, respectively. In addition, we created full-length RyR1 constructs that only contained the optimized Tc motif at the N-terminus, with or without a 10 residue histidine tag which we used for FRET measurements between FlAsH and Cy3NTA. All full length RyR1 constructs were created in the pCi-Neo mammalian expression vector (Promega, Madison, WI). The full list of constructs containing oligonucleotides, primers and methods of construction are provided in supplemental Fig. S1.

### Cell Culture

HEK-293T cells were grown and transiently transfected with RyR1 cDNAs using polyethylenimine as described previously [10]. Cells were labeled with biarsenical reagents and examined using fluorescence microscopy two days after transfection.

### Labeling with biarsenical fluorophores

FlAsH was obtained as the Lumio Green product from Life Technologies (Grand Island, NY) and ReAsH was a generous gift from Dr. Henry Paulus at the Boston Biomedical Research Institute. Labeling of RyR1 containing Tc tag fusions was conducted as described [16] with the following modifications. FlAsH or ReAsH were first complexed with ethane dithiol (EDT) for 10 min at room temperature by mixing 12.5 mM EDT and 0.5 mM FlAsH/ReAsH in 50% dimethylsulfoxide (DMSO). This reaction mixture diluted 1∶500 in FRET buffer (125 mM NaCl, 5 mM KCl, 6 mM glucose, 25 mM HEPES pH. 7.6) was then added to HEK-293T cells expressing the Tc-tagged RyR1 constructs that had been prewashed in the same buffer. Thus, the final labeling conditions for the cells were 500 nM FlAsH/ReAsH, 12.5 µM EDT, 0.1% DMSO in FRET buffer. After 2 hours at 37 C, cells were typically washed three times in FRET buffer followed by postwashing with 0.25 mM British anti-Lewisite (BAL) for 15 min to reduce nonspecific labeling. After removal of BAL, cells in FRET buffer were then imaged via epifluorescence microscopy.

### Fluorescence microscopy

HEK-293T cells expressing labeled RyR1 constructs were imaged using a Nikon 60×1.20 NA water immersion objective housed within a Nikon Eclipse TE2000-U inverted microscope. FlAsH or YFP fluorescence was recorded via illumination with a YFP cubeset consisting of a 480/30 nm bandpass excitation filter, a 505 nm dichroic mirror and a 535/40 nm bandpass emission filter (Chroma Technology, Bellows Falls, VT) using a 300 W xenon lamp housed within a Lambda DG-4 lightsource (Sutter Instruments, Novato, CA). ReAsH fluorescence was recorded via illumination with a ReAsH filter set consisting of a 570/20 nm bandpass emission filter, 585 nm dichroic mirror and a 620/60 nm emission filter (Chroma). Fluorescence was recorded using a Stanford Photonics XR-MEGA 10 camera as a series of sixty 16-bit 1344×1032 pixel images across a Z-stack 60 microns in thickness. Pseudocolored images were presented as Z-projections that depict the Z-stack's maximum fluorescence values at each image pixel, thus enabling cells at different Z-planes to be displayed in the same image. Image processing was performing using Image J v. 1.45 m (NIH).

### FRET Imaging

In some experiments, FlAsH or ReAsH was used as a FRET donor or acceptor, respectively. In these cases, FRET was quantified from the increase in donor fluorescence after selective photobleaching of acceptor for 4 min at maximum light output using the ReAsH filter set. Donor fluorescence was acquired either before or after acceptor photobleaching, and the FRET efficiency (E) was calculated using E = 1−(F_prebleach_/F_postbleach_) where F_prebleach_ and F_postbleach_ are donor fluorescence intensities before and after photobleaching of the acceptor. Donor fluorescence was quantified from background-corrected images derived from these experiments using ImageJ as described [11]. FRET experiments using 1 µM Cy3NTA as FRET acceptor were conducted as described [11].

In some cases, FRET efficiencies were converted to intramolecular distances using R = R_0_((1/E)−1)^1/6^ where R represents the donor/acceptor distance, R_0_ represents the Förster distance for the donor/acceptor pair, and E represents the measured FRET efficiency. The R_0_ values determined for the donor/acceptor pairs (calculated as described [10]) were 54.3 Å for YFP/ReAsH and 62.5 Å for GFP/Cy3NTA.

### RyR1 Immunocytochemistry

HEK-293T cells expressing FlAsH-labeled FLNRyR1 were washed three times with phosphate buffered saline (PBS; 137 mM NaCl, 2.7 mM KCl, 10 mM Na_2_HPO_4_, 1.8 mM KH_2_PO_4_, pH 7.4) followed by fixation in 0.5% formalin for 30 min at room temperature. Cells were then washed three times in PBS followed by a 30 min incubation in blocking solution consisting of 2.5% normal goat serum and 0.1% saponin. After washing, cells were treated with 34 C anti-RyR monoclonal antibody (Developmental Studies Hybridoma Bank, Iowa City, IA) [19] diluted 1∶200 for 30 min. The cells were then washed three times with PBS and then exposed for 30 min to a rhodamine-conjugated goat anti-mouse secondary antibody (Sigma, St. Louis, MO) diluted 1∶2000. After washing, cellular fluorescence was visualized at 60× magnification using cubesets for either YFP (for FlAsH) or ReAsH (for RyR1 immunocytochemistry).

## Results and Discussion

### ReAsH Labeling

**Figure 1:**
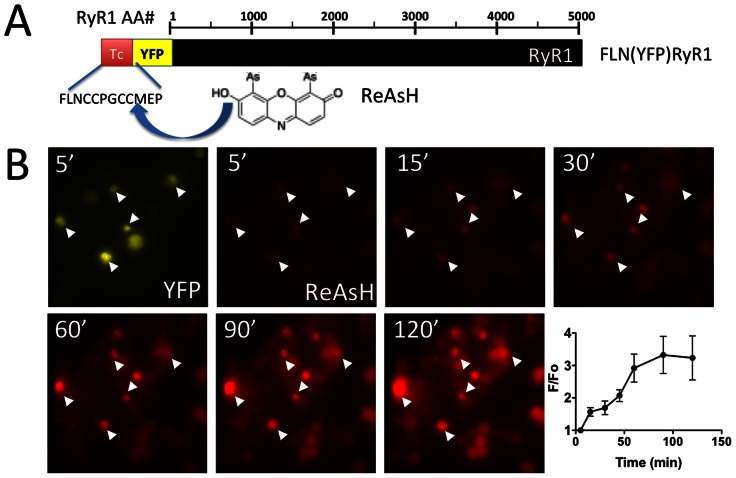
ReAsH labeling of Tc-tagged RyR1. (A) ReAsH binding to an optimized Tc tag attached to a YFP-RyR1 fusion protein (FLN(YFP)RyR1) was assessed via determination of YFP/ReAsH colocalization in intact cells. Black bar indicates RyR1 primary sequence. (B) ReAsH labeling timecourse of HEK-293T cells expressing FLN(YFP)RyR1. YFP (upper left panel) and ReAsH fluorescence (subsequent panels) was recorded at the indicated timepoints after addition of ReAsH. Arrowheads indicate same cells in all panels. ReAsH labeling timecourse is also presented as aggregate ReAsH fluorescence at each timepoint normalized to initial fluorescence 5 min after ReAsH addition (F/F_0_) (lower right).

We first tested the ability of the biarsenical compound, ReAsH to label tetracysteine tags introduced into RyR1, the skeletal muscle-specific RyR isoform [20]. We created FLN(YFP)RyR1, a RyR1 construct containing an optimized biarsenical recognition sequence, FLNCCPGCCMEP [15] directly adjacent to a bright YFP variant, citrine [18] fused to the N-terminus of RyR1 (Fig. 1A). This construct exhibited intense YFP fluorescence when expressed in HEK-293T cells (Fig. 1B). Upon addition of 500 nM ReAsH, characteristic red fluorescence developed in cells expressing FLN(YFP)RyR1 within 5 minutes but within 1 hr, ReAsH labeling of nontransfected cells was clearly evident as well (Fig. 1B). This finding was not unexpected since nonspecific labeling of intracellular proteins by biarsenical reagents has been well-documented (for example, see [21]). Labeling was essentially complete after 1 hr (Fig. 1B).

**Figure 2:**
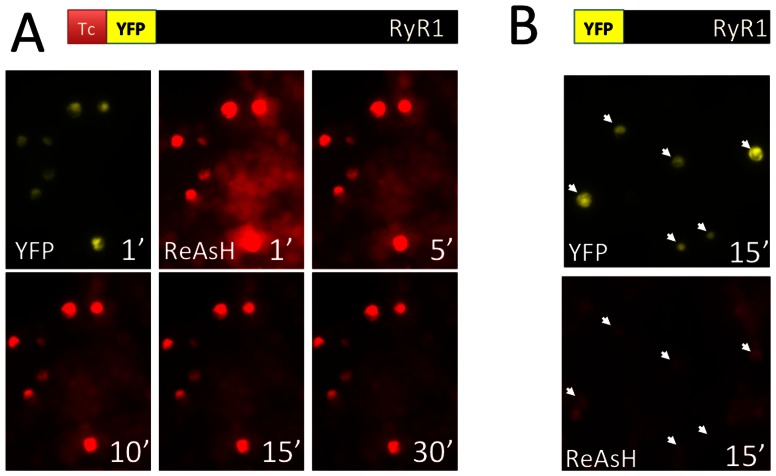
Removal of nonspecific ReAsH labeling using BAL. (A) HEK-293T cells expressing FLN(YFP)RyR1 labeled with ReAsH were incubated with 0.25 mM BAL. YFP (top left panel) or ReAsH fluorescence (subsequent panels) was recorded at the timepoints indicated after addition of BAL. Colocalization of YFP and ReAsH in these cells was plainly evident after 10′ incubation in BAL. (B) YFP (top panel) and ReAsH fluorescence (bottom) for ReAsH-treated HEK-293T cells expressing (YFP)RyR1 (which lacks a Tc tag) were recorded 15′ after treatment with 0.25 mM BAL. No specific YFP/ReAsH colocalization was observed. Arrows indicate same cells in both images in (B).

We then used the sulfhydryl reagent, British anti-Lewisite (BAL), to reduce nonspecific labeling by ReAsH, as has been reported previously [15], [17], [22]. After treatment with 0.25 mM BAL, ReAsH labeling of untransfected cells decreased rapidly whereas labeling of FLN(YFP)RyR1-expressing cells was maintained even after 30 min in BAL (Fig. 2A). This specific ReAsH labeling required the optimized Tc tag on RyR1, since no specific colocalization of ReAsH and (YFP)RyR1 (which lacks a Tc tag) was observed after treatment with BAL (Fig. 2B). Higher concentrations of BAL (>1 mM) completely removed ReAsH from the FLN(YFP)RyR1 construct (supplemental Fig. S2).

**Figure 3:**
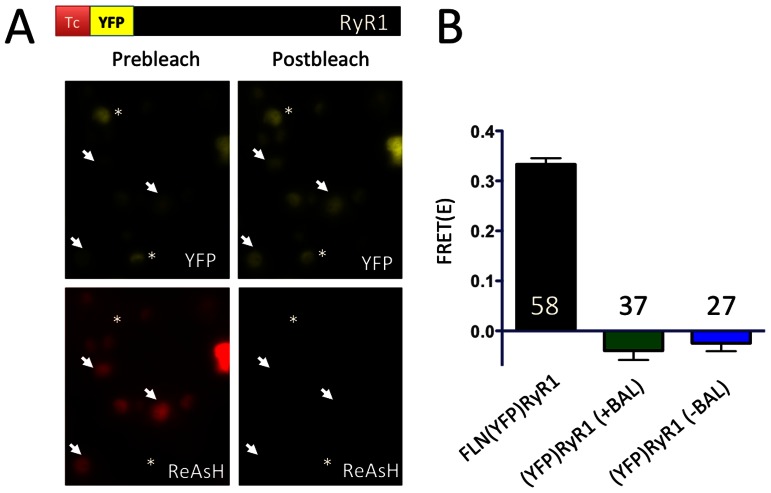
FRET measurements of FLN(YFP)RyR1 labeled with ReAsH. (A) BAL-washed HEK-293T cells expressing ReAsH-labeled FLN(YFP)RyR1 were examined for YFP (top panels) and ReAsH fluorescence (bottom panels) both before (left panels) and after (right panels) photobleaching of ReAsH. Donor fluorescence increased after acceptor photobleaching due to FRET only for those cells initially labeled with ReAsH (arrows) whereas cells that were not initially ReAsH-labeled did not display FRET (asterisks). (B) Mean FRET efficiencies are shown for FLN(YFP)RyR1 (black bar) or (YFP)RyR1 that had either been postwashed with 0.25 mM BAL for 15′ (green bar) or that was not treated with BAL (blue bar). Values represent mean +/− S.E.M. for the number of cells indicated in each bar.

To confirm specific targeting of ReAsH to the Tc tag of FLN(YFP)RyR1, we then measured energy transfer from the adjacent YFP, which acted as a FRET donor. After labeling with ReAsH and initial recording of donor and acceptor fluorescence, ReAsH was photobleached and the level of energy transfer was quantified from the resulting increase in donor fluorescence (Fig. 3A). FRET was only observed in FLN(YFP)RyR1 expressing cells labeled with ReAsH (Fig. 3A, arrows) since donor fluorescence in unlabeled cells did not increase after acceptor photobleaching (Fig. 3A, asterisks). The mean FRET efficiency observed for ReAsH-labeled FLN(YFP)RyR1 expressing cells was 0.33 whereas no FRET was observed for (YFP)RyR1 expressing cells treated with ReAsH, even when these cells were not washed with BAL (Fig. 3B). Thus, nonspecific labeling by ReAsH to non-Tc tagged sites apparently is not proximal enough to the YFP donor fused to the RyR1 N-terminus to result in FRET. This highlights an important consideration in using this reagent for FRET-based analyses: when used as a FRET acceptor, nonspecificity of labeling is not necessarily a problem as long as the nonspecific sites are far from the FRET donor. This seems to be the case, at least for the FRET donor YFP targeted to the RyR N-terminus.

ReAsH can also potentially be used in FRET experiments to measure intramolecular distances on the RyR. For example, the mean FRET efficiency value for the ReAsH-labeled FLN(YFP)RyR1 of 0.33 (Fig. 3B) corresponds to a donor/acceptor distance of 60.9 Å. In contrast, the FRET efficiency for a similar construct comprised of an N-terminal fused GFP and an adjacent His tag labeled with Cy3NTA was 0.66 [11] resulting in a donor/acceptor distance of 56 Å. Thus, even though the measured FRET efficiency values for these two systems are quite different, the measured donor-acceptor distances are remarkably consistent. The lower FRET efficiency for the YFP/ReAsH pair most likely stems from a relatively lower R_0_ value (54.3 Å for YFP/ReAsH compared to 62.5 Å for GFP/Cy3NTA [10]) and these differences in R_0_ values result from the relatively lower molar extinction coefficient for ReAsH (ε_571_ = 63,000 cm^−1^ M^−1^) [23] compared to Cy3NTA (ε_550_ = 150,000 cm^−1^ M^−1^) [10]. For longer distance measurements, acceptors can potentially be used that have higher molar extinction coefficients such as Cy3- [24] and Alexa Fluor 594-conjugated biarsenicals [25].

### Potential for orthogonal labeling of RyR1 using FlAsH and ReAsH

Zurn et. al reported that FlAsH and ReAsh could be used to label two proteins at Tc tags with varying affinities for FlAsH and ReAsH [17]. They tagged the membrane-bound parathryroid hormone receptor with the optimized dodecapeptide Tc tag, FLNCCPGCCMEP and then tagged the cytosolic beta-arrestin protein with the core CCPGCC Tc sequence. They then expressed these proteins in HEK-293 cells, labeled them with ReAsH, and washed the proteins with a high concentration of BAL, to remove ReAsH from all nonspecific sites as well as the low affinity Tc tag. They then added FlAsH, which resulted in a double-labeled system with FlAsH bound to the core Tc tag and ReAsH bound to the optimized Tc motif.

**Figure 4:**
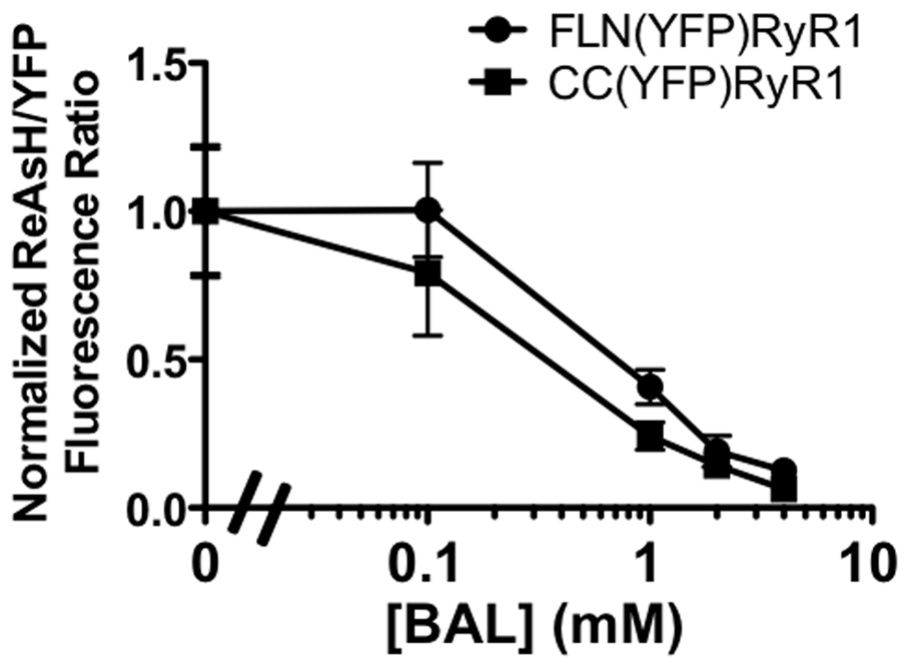
ReAsH binding stability to two different Tc tags on RyR1. HEK-293T cells expressing FLN(YFP)RyR1 or CC(YFP)RyR1 labeled with ReAsH were incubated in increasing [BAL] for 15′ followed by quantification of YFP and ReAsH fluorescence. Values represent mean ReAsH/YFP fluorescence ratio normalized to this ratio acquired at 0 BAL for 9–12 cells per datum point.

We sought to adapt this system for FRET-based analyses of RyR1. This system would enable intact cell labeling of RyR1 at two different Tc tags, thus eliminating the need to use a fused fluorescent protein as FRET donor. To test the feasibility of this approach, we constructed CC(YFP)RyR1, a YFP-tagged RyR1 construct that contained just the core Tc motif, CCPGCC. We then compared ReAsH binding stability either to this construct or to FLN(YFP)RyR1 (which contains the optimized Tc tag) by incubating these labeled cells in increasing concentrations of BAL and then measuring the relative ReAsH/YFP fluorescence ratio (for images, see supplemental Fig. S2 and S3). For both constructs, specific labeling began to decrease at 1 mM BAL whereas at 4 mM BAL, ReAsH had been almost entirely removed (Fig. 4). Thus, no apparent difference in ReAsH binding stability was evident between the optimized and non-optimized Tc tags on RyR1. It is unclear why this is the case although presumably the flanking sequences around the core CCPGCC motif in our nonoptimized Tc tag on RyR1 may play a role. Other reported Tc tag recognition sequences with differential affinities for FlAsH and ReAsH such as CCKACC [26] may prove useful in developing this type of double labeling system for future FRET-based studies of the RyR.

### FlAsH Labeling

**Figure 5:**
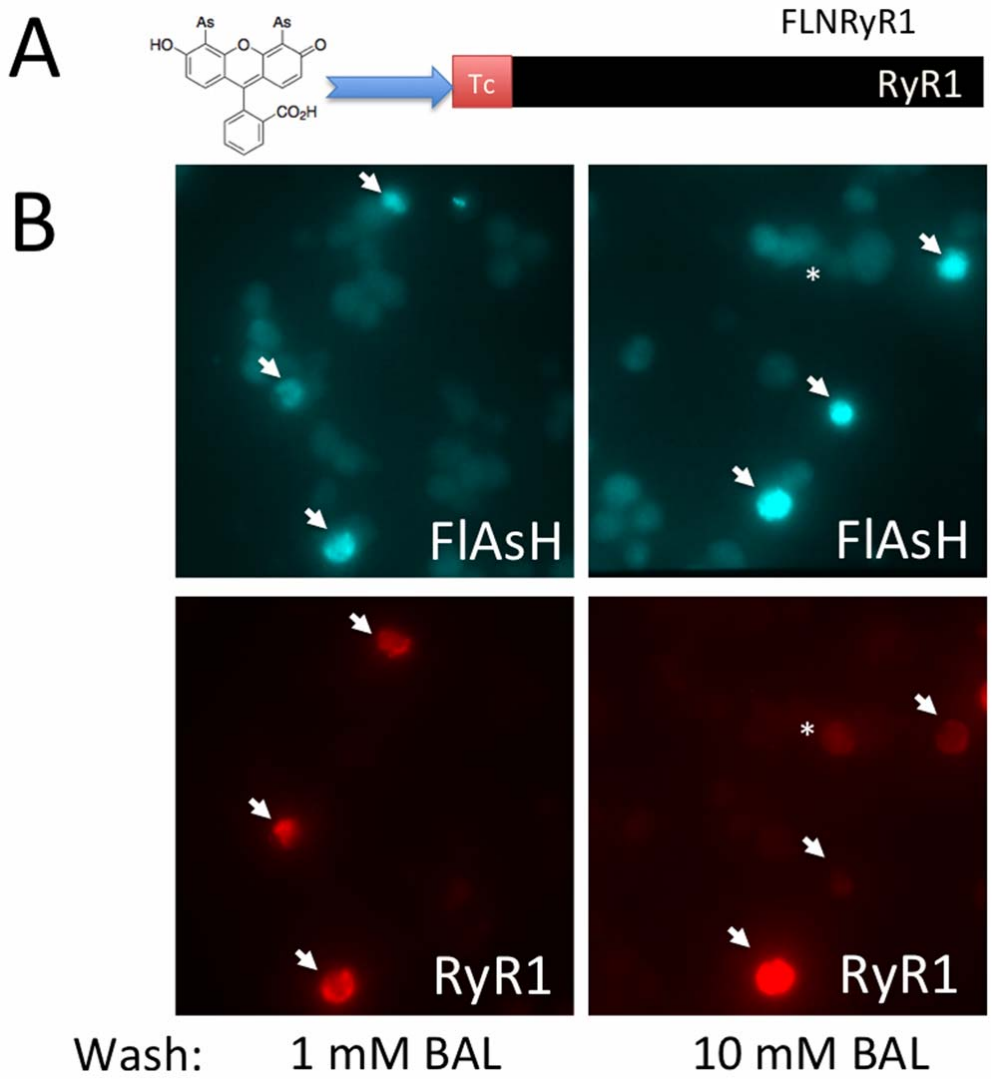
FlAsH labeling of Tc-tagged RyR1. (A) Scheme for FlAsH labeling of RyR1 containing an optimized tetracysteine tag at the N-terminus (FLNRyR1). (B) Cells expressing FLNRyR1 were labeled with FlAsH and then washed with either 1 mM (left panels) or 10 mM BAL (right panels). Panels depict either the FlAsH label (top panels) or RyR1 detected via immunocytochemical analysis using the 34 C anti-RyR antibody (bottom panels). Arrows depict same cells in both the top and bottom panels of each set.

Next we explored the possibility of using the fluorescein-derived biarsenical reagent, FlAsH to label RyR1. FlAsH has very similar spectral characteristics compared to YFP, so theoretically, it could act as a donor in FRET experiments with our His-tag selective FRET acceptor, Cy3NTA. To test this approach, we first created FLNRyR1, which contained the optimized Tc sequence (FLNCCPGCCMEP) at the N-terminus of RyR1 (Fig. 5A). We expressed this construct in HEK-293T cells and then labeled it with 0.5 µM FlAsH followed by postwashing with either 1 mM or 10 mM BAL (Fig. 5B). Though some preferential FlAsH labeling of these cells was evident in these experiments (arrows), most cells were still labeled with FlAsH even after washing with 10 mM BAL. The bright FlAsH labeling often colocalized with anti-RyR immunocytochemistry (arrows) though some RyR expressing cells were not preferentially labeled with FlAsH (asterisk) (Fig. 5B).

**Figure 6:**
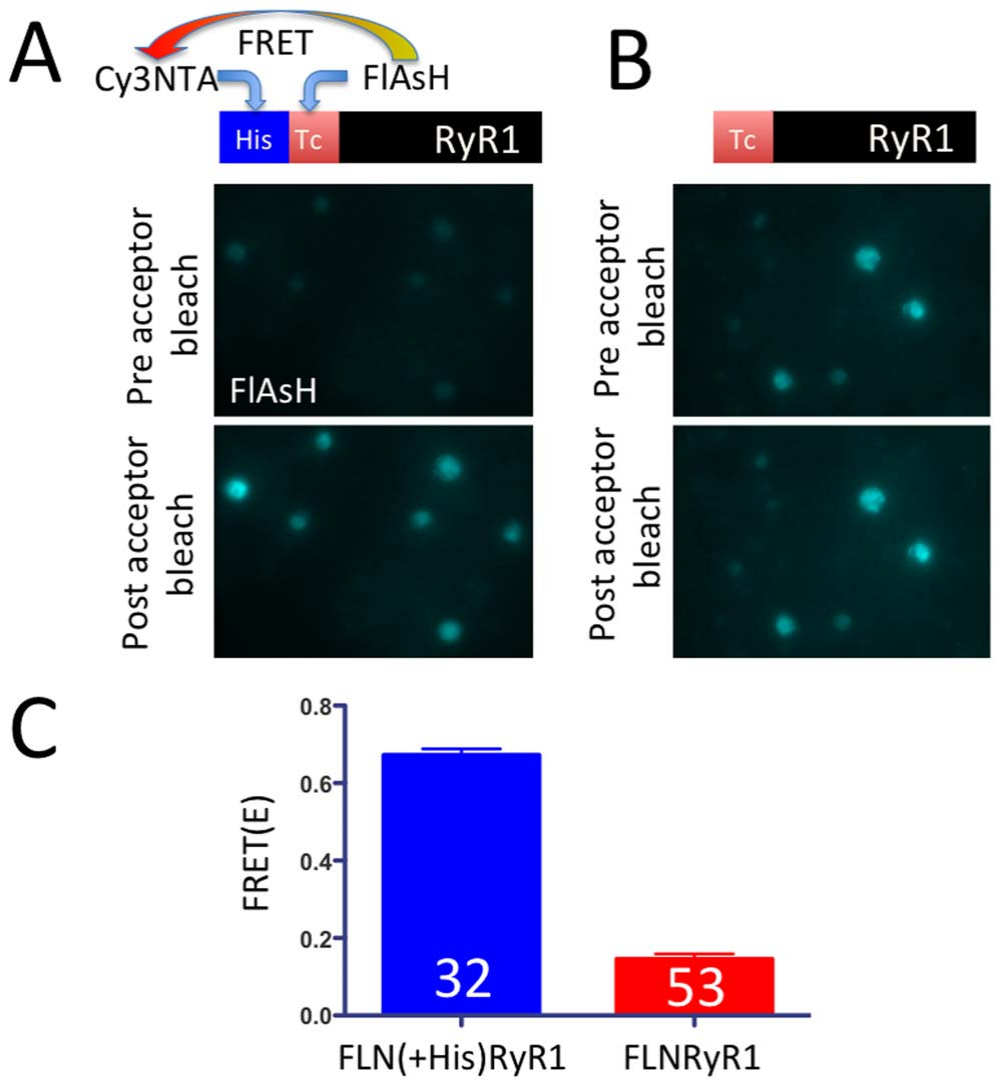
FRET analysis of FlAsH-labeled RyR1 constructs. HEK-293T cells expressing either FLN(+His)RyR1 (A) or FLNRyR1 (B) were labeled with FlAsH (acting as FRET donor) and Cy3NTA (FRET acceptor). FlAsH fluorescence was recorded before (top panels) and after (bottom panels) photobleaching of the Cy3NTA acceptor. FRET was observed as an increase in FlAsH fluorescence after acceptor photobleaching. (C) Mean FRET efficiencies are shown for FlAsH-labeled FLN(+His)RyR1 and FLNRyR1 using Cy3NTA as FRET acceptor. Values represent mean +/− S.E.M for the number of cells indicated in each bar.

We then tested the ability of FlAsH targeted to RyR1 to act as a FRET donor. We fused a His_10_ tag binding site for the FRET acceptor, Cy3NTA to the N-terminus of FLNRyR1 to create FLN(+His)RyR1 (Fig. 6A). After labeling this construct with both FlAsH and Cy3NTA, donor fluorescence was recorded both before and after photobleaching of the CyNTA acceptor (Fig. 6A). We observed a high degree of energy transfer (E = 0.67) for Cy3NTA targeted to FlAsH-labeled FLN(+His)RyR1 (Fig. 6C), thus indicating successful targeting of both FlAsH and Cy3NTA to orthogonal tags fused to the N-terminus of the ryanodine receptor. In contrast, markedly lower levels of energy transfer (E = 0.14) were observed between FlAsH and Cy3NTA targeted to FLNRyR1, which lacks a His tag (Fig. 6B,C).

These experiments document the first combined use of the Tc tag and His tag labeling systems, to our knowledge. However, several problems exist with the use of FlAsH. Similar to ReAsH, this compound also labels intracellular proteins nonspecifically but we were not able to remove all nonspecific-bound FlAsH, even with BAL concentrations as high as 10 mM. Upon permeabilization of the cells, much of the nonspecific FlAsH labeling was removed, which suggests that the compound labels soluble proteins in the cell, which are lost after permeabilization. In addition, FlAsH appears to only label a subset of cells expressing the Tc-tagged RyR1. It is not clear why this is the case since FlAsH is a membrane-permeable compound that clearly labels most cells even if much of this labeling is nonspecific. However, this property does not necessarily limit its use in FRET studies since it is being used as a FRET donor and thus, analysis can be confined to just the labeled cells.

### Comparison with existing labeling systems

Our interest in using biarsenical compounds to label the ryanodine receptor stems from the need for a novel orthogonal labeling strategy to overcome deficiencies in current labeling methods used for FRET studies. For example, fluorescent protein (FP) fusions have been used to target both donor and acceptor fluorophores to the ryanodine receptor [6], [7], [8]. While these proteins can be inserted with essentially 100% labeling efficiency, their bulky size can disrupt the native protein structure or function, which in turn limits the number of potential insertion sites. In addition, the lengthy glycine-rich linkers used to tether these proteins to the RyR as well as the physical dimensions of the FPs themselves can add significant distances from the insertion site to the center of the FP fluorophore [11], which hampers the interpretation of FRET-based distance measurements. In contrast, fluorescently labeled RyR-associated proteins used as FRET donors and acceptors [3], [4], [5] bind to well-characterized, physiological sites on the native RyR. However, these associated proteins require permeabilization of the cells and their versatility is limited to just their physiological binding sites on the RyR. The final method used to tag the RyR with fluorophores for FRET studies is our His tag labeling system using Cy3NTA, which we have used to label numerous locations throughout the ryanodine receptor [10], [11]. However, this method requires permeabilization of the cells in order for Cy3NTA to enter, which prevents the use of this technique in applications that require an intact cell membrane, such as measuring changes in RyR structure during EC coupling. In addition, Cy3NTA binding to the His tag is noncovalent, and thus the compound may wash off during perfusion of the RyR with agonists and antagonists during dynamic FRET measurements.

The biarsenical-based approach developed in this report solves many of these problems. Using the YFP/ReAsH FRET system, we can label RyR1 with ReAsH and then perform FRET measurements in intact cells. In addition, the binding of biarsenicals is covalent, and so these compounds should remain attached to their Tc binding sites on RyR1, even during perfusion experiments. Finally, for the FlAsH/Cy3NTA system, no fluorescent proteins are required, thus enabling FRET experiments to be performed using two completely distinct site-specific labeling systems, thereby affording unprecedented flexibility in sites for FRET measurements.

### Summary

In summary we have demonstrated successful labeling of Tc motifs with biarsenical fluorophores for FRET-based structural determinations of the ryanodine receptor. The use of these methods provides a powerful new system that can be used in conjunction with previously established techniques for future FRET-based analyses of the RyR.

## Supporting Information

Figure S1 **Detailed methods for introducing Tc tags into RyR1 are described.**
(DOCX)

Figure S2 **ReAsH binding stability to optimized Tc tag,** **FLNCCPGCCMEP** **.** ReAsH-labeled HEK-293T cells expressing FLN(YFP)RyR1 were incubated in the indicated concentrations of BAL for 15′. YFP (top images) or ReAsH fluorescence (bottom) was then recorded. The relative YFP/ReAsH fluorescence ratio was then determined and plotted at each BAL concentration (Fig. 4).
(TIF)

Figure S3 **ReAsH binding stability to core Tc tag,** **CCPGCC** **.** ReAsH-labeled HEK-293T cells expressing CC(YFP)RyR1 were incubated in the indicated concentrations of BAL for 15′. YFP (top images) or ReAsH fluorescence (bottom) was then recorded. The relative YFP/ReAsH fluorescence ratio was then determined and plotted at each BAL concentration (Fig. 4).
(TIF)
