# Supplementary material for: Site-Specific Labeling of the Type 1 Ryanodine Receptor Using Biarsenical Fluorophores Targeted to Engineered Tetracysteine Motifs
Source: PLoS One. 2013 May 28;8(5):e64686. doi: 10.1371/journal.pone.0064686 (PMC3665623; doi:10.1371/journal.pone.0064686)
Supplement: Figure S1 — Detailed methods for introducing Tc tags into RyR1 are described. (DOCX) [file pone.0064686.s001.docx]

**Supplemental Figure S1: Molecular cloning methods.**

cDNA encoding tetracysteine (Tc) tags were introduced into RyR1 as follows. First, a Tc tag was inserted into the N-terminus of GFP via PCR primer extension using:

5’-**CCATGG**ATTTCT**CAATTG**TTGCCCAGGCTGTTGCATGGAAC*CCGGAATGGTGAGCAAG****GGCGCC****GCG*-3’

where bold nucleotides indicate NcoI, MfeI, and SfoI sites used for cloning, underlined residues encode the FLNCCPGCCMEP Tc tag and italicized residues annealed to the GFP cDNA.

To substitute the cDNA for YFP (citrine) into this clone, YFP cDNA was PCR-amplified using:

5’-**GGCGCC**GAGCTGTTCACCGGGGTGG-3’ and

5’-**GCGGCCGC**TTACTTGTACAGCTCGTCCATGCCG-3’

to introduce SfoI and NotI sites (in bold) used to substitute YFP into the FLN-GFP construct to create **FLN-YFP**. This clone was then amplified with PCR primers containing SanD1 restriction sites (in bold):

5’-**GGGTCCC**GCGGGCCTCGACATCATGGGCAGCAGCCATCATCATCATCATCACC-3’

5-**GGGACCC**CCACCTCCACTACCTCCACCTCCCTTGTACAGCTCGTCCATGCC-3’

and the resulting clone was digested with SanD1 and substituted into full length GFP-RyR1 to create **FLN(YFP)RyR1** used in Figures 1-4.

To create **(YFP)RyR1** (Fig. 3), the Tc tag cDNA was excised from FLN(YFP)RyR1 using SacII and EcoRV and a small oligo was inserted (upper primer only is shown, start codon in bold):

5’-GGGCCTCGACATC**ATG**GGATCGGAT-3’

To create **CC(YFP)RyR1** (Fig. 4) (YFP)RyR1 was digested with Sac II and EcoRV and the following oligo was inserted (upper primer only is shown, start codon bold, Tc tag underlined):

5’-GGGCCTCGACATC**ATG**GGCTGTTGCCCTGGTTGTTGCGAT-3’

**FLNRyR1** (Fig. 5,6) was created via digestion of FLN(YFP)RyR1 with SanD1 (which excises YFP) and substitution of the following oligo (upper primer only is shown, start codon bold, Tc tag underlined):

5’- GTCCCGCGGGCCTCGACATC**ATG**GGCTTTCTCAATTGTTGCCCAGGCTGTTGCATGGAACCCGCGG -3’

Finally, a 10 residue His tag was attached to the N-terminal end of FLNRyR1 using an oligo that contained a Sac II 5’ sticky end and an Mfe I 3’ sticky end with the following sequence:

5’- GGGCCTCGACATC**ATG**GGCCATCACCATCACCATCACCATCACCATCACTTTCTC-3’

where the start codon is in bold and the His tag is encoded by the underlined sequence. This oligo was substituted into FLNRyR1 to create **FLN(His)RyR1** used in Figure 6.
