# Supplementary figures and images for: Site-Specific Labeling of the Type 1 Ryanodine Receptor Using Biarsenical Fluorophores Targeted to Engineered Tetracysteine Motifs
Source: PLoS One. 2013 May 28;8(5):e64686. doi: 10.1371/journal.pone.0064686 (PMC3665623; doi:10.1371/journal.pone.0064686)

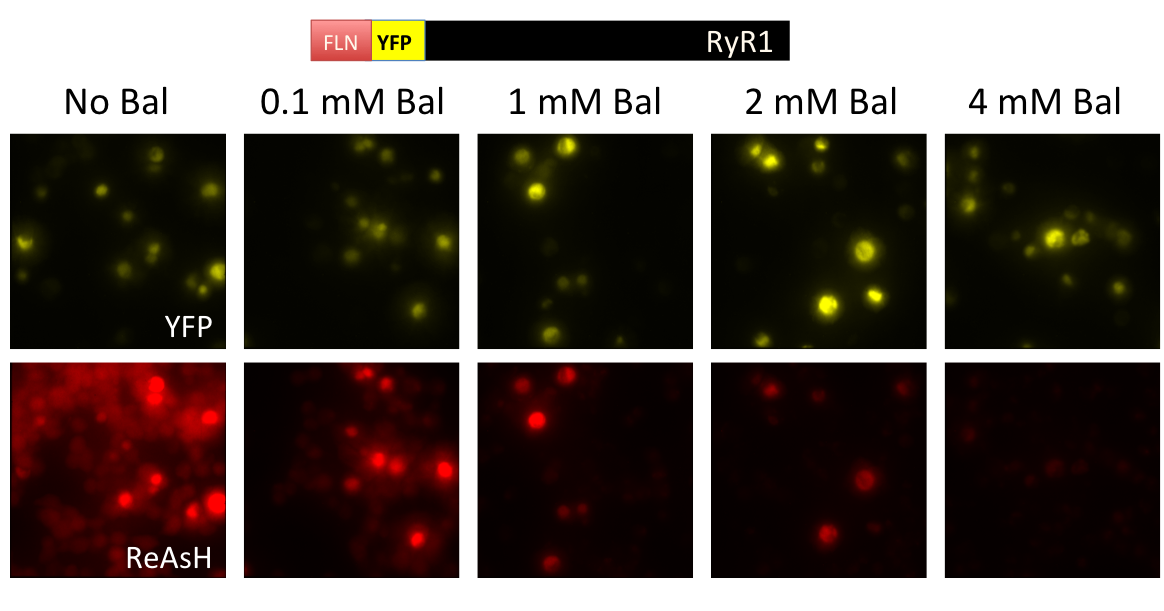

Supplement: Figure S2 — ReAsH binding stability to optimized Tc tag, FLNCCPGCCMEP . ReAsH-labeled HEK-293T cells expressing FLN(YFP)RyR1 were incubated in the indicated concentrations of BAL for 15′. YFP (top images) or ReAsH fluorescence (bottom) was then recorded. The relative YFP/ReAsH fluorescence ratio was then determined and plotted at each BAL concentration (Fig. 4). (TIF) [file pone.0064686.s002.tif]

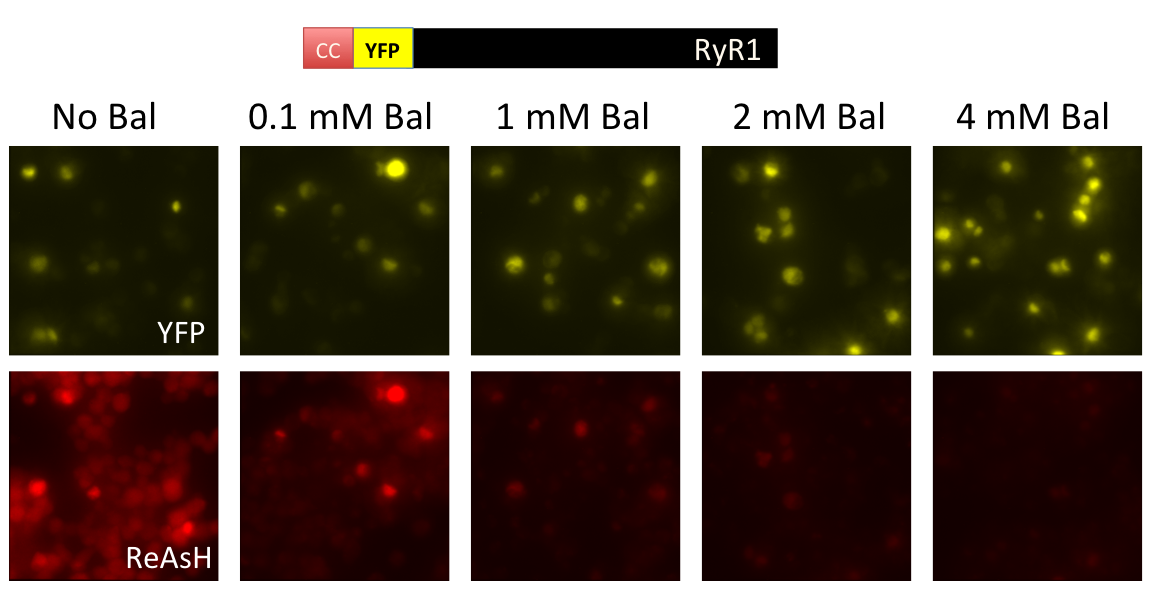

Supplement: Figure S3 — ReAsH binding stability to core Tc tag, CCPGCC . ReAsH-labeled HEK-293T cells expressing CC(YFP)RyR1 were incubated in the indicated concentrations of BAL for 15′. YFP (top images) or ReAsH fluorescence (bottom) was then recorded. The relative YFP/ReAsH fluorescence ratio was then determined and plotted at each BAL concentration (Fig. 4). (TIF) [file pone.0064686.s003.tif]
